# A double patella-like condition secondary to synovial osteochondromatosis

**DOI:** 10.1186/1758-2555-4-31

**Published:** 2012-09-03

**Authors:** Yoshiteru Kajikawa, Yuji Arai, Hisatake Takamiya, Tetsuo Higuchi, Gen Mori, Shinsuke Morisaki, Toshikazu Kubo

**Affiliations:** 1 Department of Orthopaedics, Graduate School of Medical Science, Kyoto Prefectural University of Medicine, Kawaramachi Hirokoji, Kamigyo-ku, Kyoto, 602-8566, Japan

**Keywords:** Osteochondromatosis, Synovial osteochondromatosis, Chondromatosis, Double patella, Knee

## Abstract

To our knowledge, this is the first case of synovial osteochondromatosis in a patient presenting with a double patella-like condition. The true duplication of the patella, which is called double patella, is extremely rare. In our case, the operative and histopathological findings showed that the double patella-like condition was secondarily induced by synovial osteochondromatosis. Synovial osteochondromatosis should be considered as a differential diagnosis for congenital double patella.

## Background

True duplication of the patella, which is called double patella, is extremely rare [1]. To our knowledge, the total number of reported cases is only 15. When a case with double patella is examined, bipartite patella, patella fractures, and postoperative morphological changes should be considered in differential diagnosis.

## Case presentation

**Figure 1:**
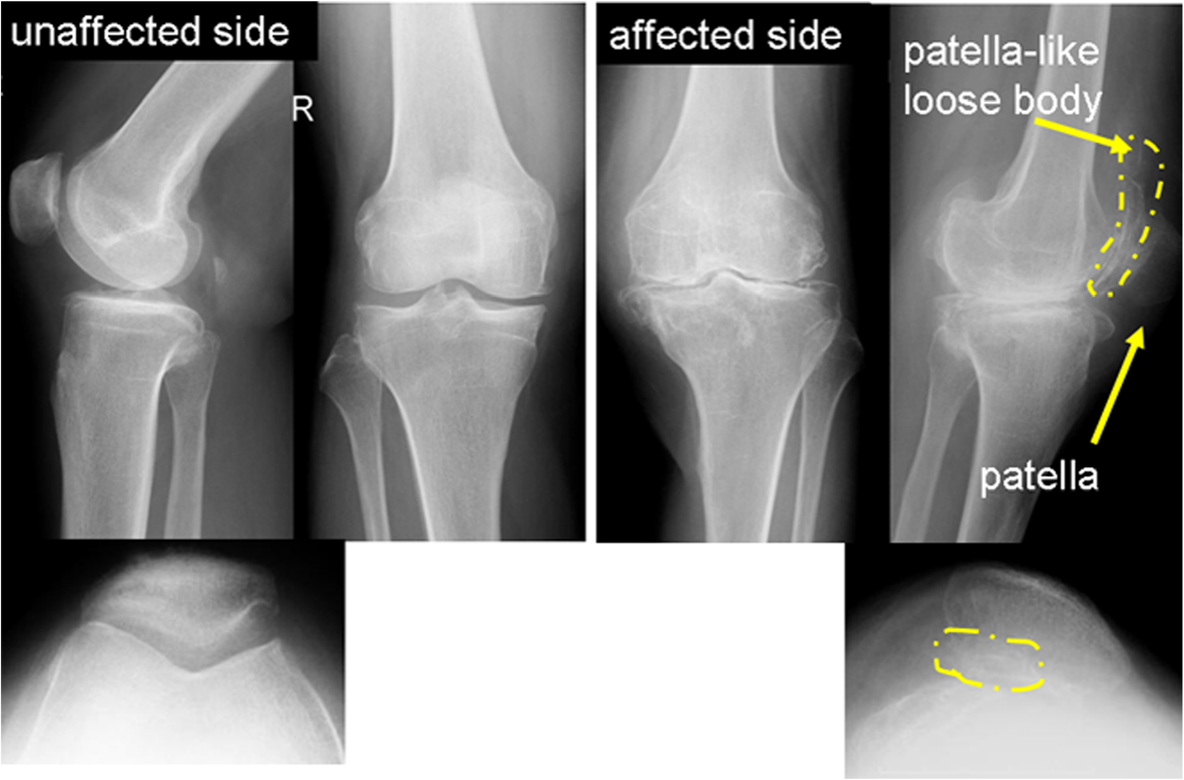
**X-ray analysis.** X-ray analysis showed a bone fragment in the patellofemoral joint.

**Figure 2:**
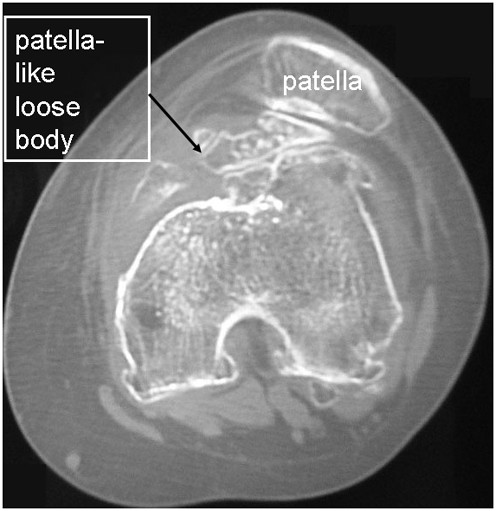
**CT.** The left knee had formed a double-layered patella.

**Figure 3:**
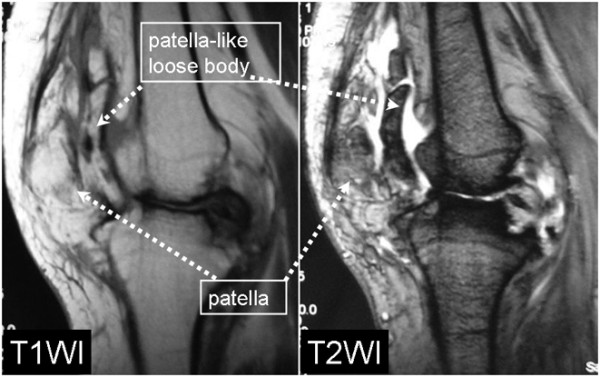
**MRI.** T2-weighted MRI showed a high-signal-intensity area around the bone fragment, and thus, the fragment was considered to be an intraarticular loose body.

**Figure 4:**
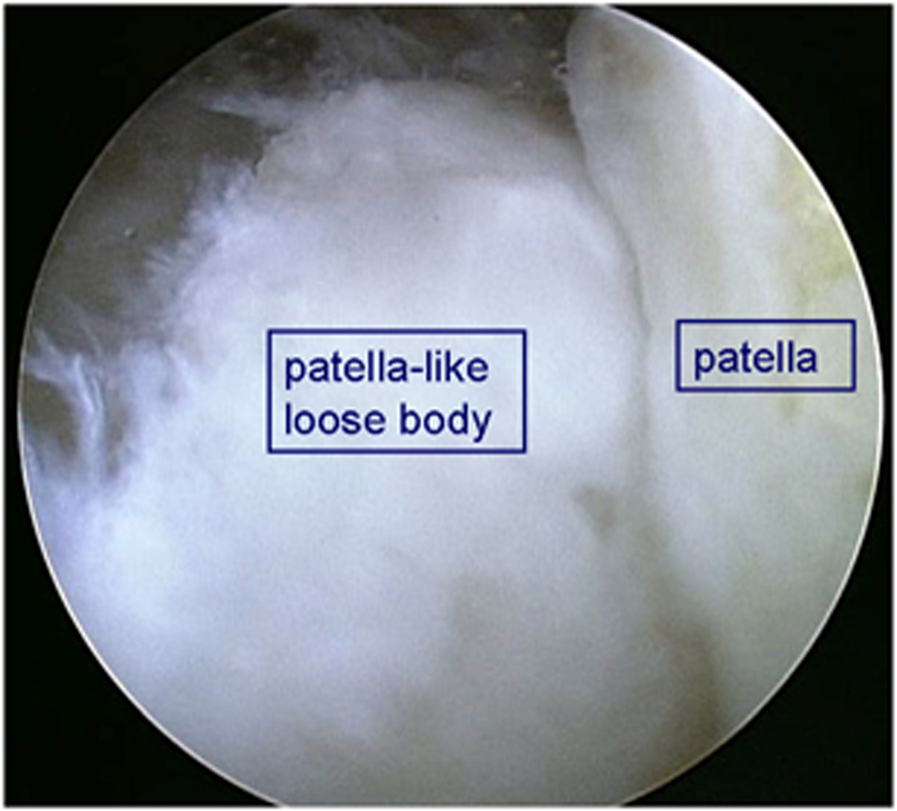
**Arthroscopy.** Arthroscopic findings showed that the loose body was completely covered with cartilage-like tissue. The loose body was connected by white fibrous tissue to the lateral plica synovialis, but it did not have bony continuity with the patella or femur.

**Figure 5:**
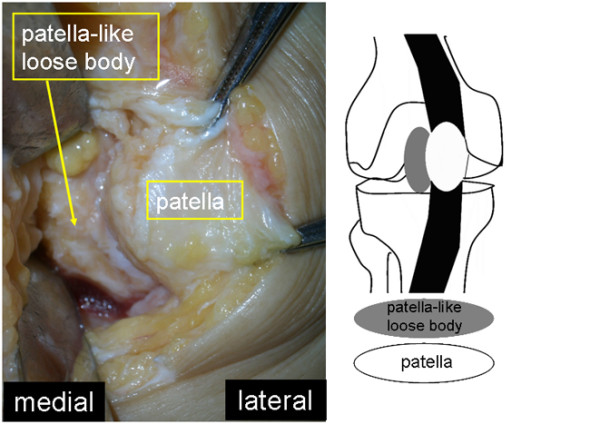
**Resection of the loose body.** We took a medial parapatellar approach and resected the loose body.

**Figure 6:**
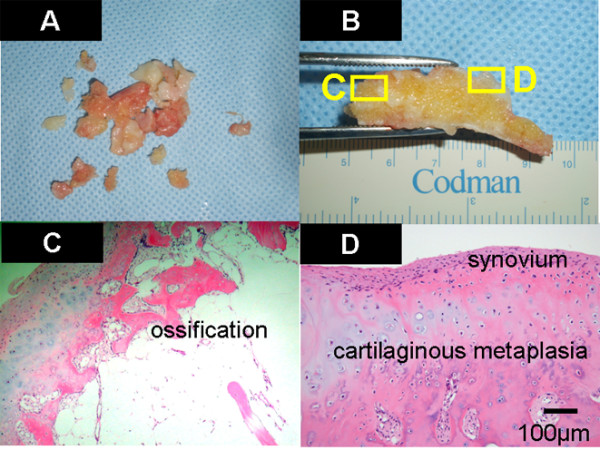
**Histopathological findings.** Many smaller loose bodies were excised at the same time (Figure 6**A**). Histopathological findings showed that the loose bodies were almost completely covered with synovial membrane. The loose bodies were cartilaginous, and cartilaginous metaplasia was observed in the area where the synovial membrane covered the loose body (Figure 6**B**, **D**). The cartilage showed proliferative activity with large pleomorphic nuclei, which tended to be arranged in small bunches. Furthermore, ossification was partially found in the loose body (Figure 6**C**).

A 62-year-old woman had a 30-year history of pain in the left knee, which had not been treated before she was referred to our hospital. When she was 2 years old, her left knee was treated because of a suspicion of septic arthritis. She did not have operative treatment on her left knee. Although the details were unknown, she had not had any complications, such as limited range of motion (ROM) or pain in her left knee. She had pain in the left knee at the time of presentation, and she sometimes had locking in that knee. Biochemical tests showed that C-reactive protein levels and white blood cell counts were normal. Local signs of joint inflammation, tenderness, swelling, local heat, and patellar ballottement were not found. The examination showed 25° flexion and 0° extension of the left knee and 140° flexion and 0° extension of the right knee. The right knee score (Knee Society rating system) was 80/100 and left knee score was 49. X-ray analysis, including computed tomography (CT), showed a bone fragment in the patellofemoral joint and it seemed that the left knee had formed a double-layered patella (Figures 1, 2). Osteoarthritis was observed in both knees (Kellgren–Lawrence grading scale: right knee, Grade 1; left knee, Grade 3). T2-weighted magnetic resonance imaging (MRI) showed a high-signal-intensity area around the bone fragment, and thus, the fragment was considered to be an intraarticular loose body, which had neither dupricated patellar tendon nor quadiceps tendon (Figure 3). Arthroscopy and resection of the intraarticular loose body were performed. Arthroscopic findings showed that the loose body was completely covered with cartilage-like tissue. The loose body was connected by white fibrous tissue to the lateral plica synovial is, but it did not have bony continuity with the patella or femur (Figure 4). After the arthroscopy, we took a medial parapatellar approach and resected the loose body (Figure 5). In addition, we found many smaller loose bodies and excised them at the same time (Figure 6A). Histopathological findings showed that the loose bodies were almost completely covered with synovial membrane, which formed synovial nodules. The synovial membrane connected the loose body to the lateral plica synovial is and articular capsule. The loose bodies were cartilaginous, and cartilaginous metaplasia was observed in the area where the synovial membrane covered the loose body (Figure 6B, D). The cartilage showed proliferative activity with large pleomorphic nuclei, which tended to be arranged in small bunches. Furthermore, ossification was partially found in the loose body (Figure 6C). Histological examination of the synovial osteochondromatosis was performed on the basis of these findings. By 6 months after the operation, the patient had regained an ROM of 0° to 50° without locking. The left knee score was 60/100.

## Discussion

Congenital duplication of the patella is called double patella [1]. To our knowledge, double patella is extremely rare, and the total number of reported cases is only 15.

When a case with double patella is examined, bipartite patella, patella fractures, and postoperative morphological changes should be considered in differential diagnosis. Bipartite patella is more common than double patella. It has been hypothesized that bipartite patella is due to the failure of the ossification nuclei to merge. The presence of 2 distinct and separate ossification centers may be the cause of double patella. Double patella is a congenital malformation involving 2 independent cartilaginous centers that each give rise to a patella, complete with its own aponeurotic expansion and patella tendon [2]. MRI is very important to accurately evaluate the anatomical aspects and confirm the diagnosis of a double patella [1]. The available literature showed that in the majority of cases with double patella, the position of the second patella is superior or inferior compared with that of the normal patella, although the position of the second patella in relation to the first is unimportant. In some cases with congenital double patella, an association between the formation of double-layer patella, which is the same type as ours, and multiple epiphyseal dysplasia was described [3,4]. Only 1 isolated case of traumatic double patella has been reported [4]. Furthermore, a case with unusual double patella, which had been formed despite surgical treatment of a patella sleeve fracture, has been reported [5]. However, in these cases, the possibility of congenital double patella could be excluded on the basis of medical history and radiographic features.

In our case, the histological findings showed the features of synovial osteochondromatosis. In addition, the multifocality of small intraarticular loose bodies was consistent with this feature. We found the sites of active cartilaginous metaplasia in the synovial membrane on the patella-like loose body. Transitional lesions with both active intrasynovial proliferation and free loose bodies were confirmed, and, thus this case was diagnosed as second-phase synovial osteochondromatosis [6]. However, it is extremely rare for synovial osteochondromatosis to grow to the size of the patella, as in this case. It has been recently reported that the expression of the fibroblast growth factor (FGF) receptor was observed specifically in synovial osteochondromatosis [7]. FGFs may cause proliferative changes, such as osteophytes in osteoarthritis [8]. Although the cause of synovial osteochondromatosis is still controversial, it could be speculated that the cytokines induced by osteoarthritis, such as FGFs, activated the matrix synthesis of synovial osteochondromatosis in the patellofemoral joint. Thus, synovial osteochondromatosis should be considered as a differential diagnosis for congenital double patella.

## Conclusions

To our knowledge, this is the first case of synovial osteochondromatosis in a patient presenting with a double patella-like condition. Synovial osteochondromatosis should be considered as a differential diagnosis for congenital double patella.

## Consent

Written informed consent was obtained from the parent of the patient for publication of this case report and accompanying images. A copy of the written consent is available for review by the Editor-in-Chief of this journal.

## Competing interests

The authors declare that they have no competing interests.

## Authors’ contributions

All authors co-wrote the paper and discussed the results and commented on the manuscript. All authors read and approved the final manuscript.
